# METTL14 promotes apoptosis of spinal cord neurons by inducing EEF1A2 m6A methylation in spinal cord injury

**DOI:** 10.1038/s41420-021-00808-2

**Published:** 2022-01-10

**Authors:** Gang Gao, Yufen Duan, Feng Chang, Ting Zhang, Xinhu Huang, Chen Yu

**Affiliations:** 1 Department of spinal minimally invasive surgery, Shanxi Provincial People’s Hospital, No.29 Shuangtasi Street, Taiyuan City, Shanxi Province 030012 China; 2 Department of endocrinology, Shanxi coal central hospital, No.101 Xuefu street, Xiaodian District, Taiyuan City, Shanxi Province China

**Keywords:** Cell death in the nervous system, Spinal cord diseases

## Abstract

Spinal cord injury (SCI) is a devastating traumatic condition. METTL14-mediated m6A modification is associated with SCI. This study was intended to investigate the functional mechanism of RNA methyltransferase METTL14 in spinal cord neuron apoptosis during SCI. The SCI rat model was established, followed by evaluation of pathological conditions, apoptosis, and viability of spinal cord neurons. The neuronal function of primary cultured spinal motoneurons of rats was assessed after hypoxia/reoxygenation treatment. Expressions of EEF1A2, Akt/mTOR pathway-related proteins, inflammatory cytokines, and apoptosis-related proteins were detected. EEF1A2 was weakly expressed and Akt/mTOR pathway was inhibited in SCI rat models. Hypoxia/Reoxygenation decreased the viability of spinal cord neurons, promoted LDH release and neuronal apoptosis. EEF1A2 overexpression promoted the viability of spinal cord neurons, inhibited neuronal apoptosis, and decreased inflammatory cytokine levels. Silencing METTL14 inhibited m6A modification of EEF1A2 and increased EEF1A2 expression while METTL14 overexpression showed reverse results. EEF1A2 overexpression promoted viability and inhibited apoptosis of spinal cord neurons and inflammation by activating the Akt/mTOR pathway. In conclusion, silencing METTL14 repressed apoptosis of spinal cord neurons and attenuated SCI by inhibiting m6A modification of EEF1A2 and activating the Akt/mTOR pathway.

## Introduction

Spinal cord injury (SCI) is a kind of severe damage to the central nervous system that causes heavy physical and psychological burdens on patients worldwide [1]. The annual incidence of SCI rises to 40-80 per million people [2]. SCI can be classified into traumatic SCI and non-traumatic SCI [3]. Traumatic SCI is attributed to a direct and immediate mechanical insult (including motor vehicle collisions, falls, violence, and sports-related injuries) to the spinal cord, while non-traumatic SCI arises from insufficient blood flow, spinal tumors, and osteoarthritis [4–6]. The pathophysiology of SCI comprises ischemia, oxidative stress, inflammation, apoptosis, and locomotor dysfunctions [7]. Importantly, SCI is accepted as an incurable disease [8]. Therefore, it is imperative to understand the pathophysiology of SCI to develop therapeutic approaches.

N6-methyl-adenosine (m6A) methyltransferases are multi-component methyltransferase complexes [9]. The participation of m6A modification has been found in physiological and pathological conditions such as obesity, immuno-regulation, and carcinogenesis [10]. METTL14 (methyltransferase-like 14), a writer for m6A methylation, is an essential component to facilitate RNA binding [11]. METTL14 can suppress the differentiation of hematopoietic stem/progenitor and promote leukemogenesis through mRNA m6A modification [12]. METTL14 also promotes endothelial inflammation and atherosclerosis via inducing FOXO1 m6A modifications [13]. Moreover, METTL14 exerts important effects on maintaining striatal function and learning in mice [14]. However, the effects of METTL14 on SCI have been scarcely reported.

Eukaryotic protein elongation factor 1 alpha 2 (EEF1A2) is the second most abundant protein and an essential part composing translation machinery [15]. The loss of EEF1A2 could lead to motoneuron degeneration [16]. Purified METTL21B can methylate purified EFF1A2 in vitro [17]. Therefore, we speculated that METTL14 could mediate m6A methylation of EEF1A2. The Akt/mTOR pathway is associated with the regenerative ability of peripheral nerves [18]. METTL13 knockdown reduces phosphorylation levels of AKT and mTOR [19]. EEF1A2 protects against apoptotic cell death by activating the PI3K/Akt pathway [20]. However, it remains unknown whether METTL14 could mediate m6A methylation of EEF1A2 and regulate the Akt/mTOR pathway in SCI. This study explored the functional mechanisms of METTL14 in neuron apoptosis following SCI by inducing m6A methylation of EEF1A2 and modulating the Akt/mTOR pathway.

## Results

### EEF1A2 was poorly expressed and Akt/mTOR pathway was inhibited in SCI

**Fig. 1:**
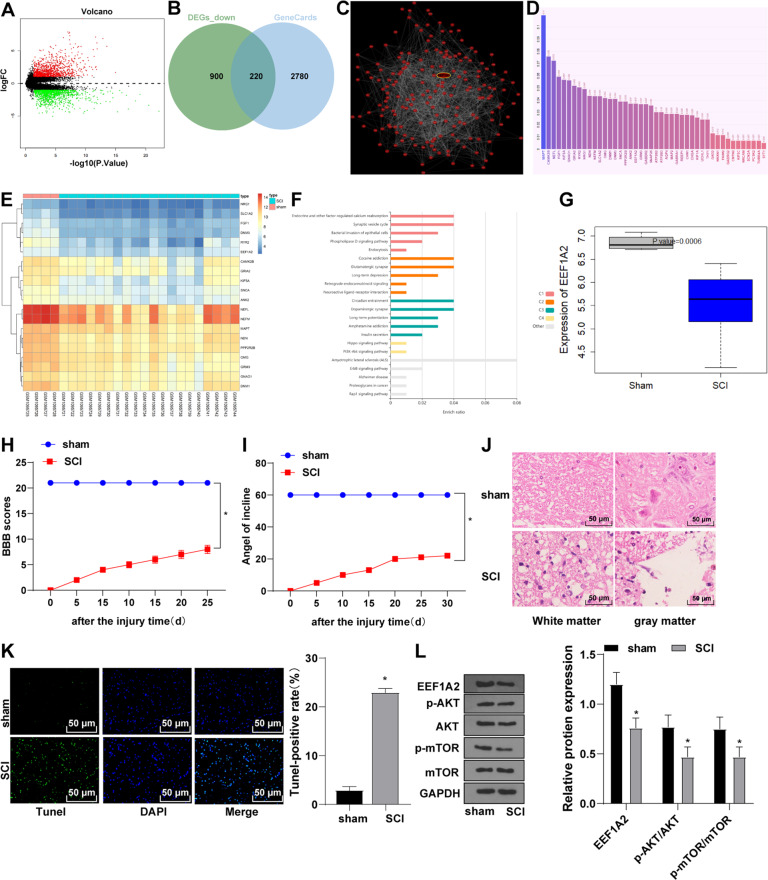
EEF1A2 was poorly expressed and Akt/mTOR pathway was inhibited in SCI. **A** differential expression gene map of SCI chip GSE45006, the abscissa represents –log (*p* value), the ordinate represents log2FC, red dots indicate highly-expressed genes and green dots indicate lowly-expressed genes; (**B**) Venn map of the intersection of differentially low-expression genes and GeneCards database; (**C**) gene co-expression network by Coexpedia; (**D**) bar graph of the correlation of gene-disease, the abscissa represents correlation score; (**E**) expression heatmap of candidate genes, the color scale represents gene expression from low (blue) to high (red); (**F**) enriched terms visualized in barplot, each row represents an enriched function, and the length of the bar represents the enrich ratio, which is calculated as “input gene number”/“background gene number”. The color of the bar is the same as the color in the circular network in above, which represents different clusters. For each cluster, if there are more than five terms, top 5 with the highest enrich ratio will be displayed; (**G**) significant low expression of EEF1A2 in chip GSE45006; (**H**) locomotor ability of rats detected by BBB locomotor rating scale; (**I**) neuronal function detected by inclined plane test; (**J**) pathological conditions of spinal cord assessed by HE staining; (**K**) apoptosis of spinal cord tissues detected by TUNEL staining; (**L**) expressions of EEF1A2 and Akt/mTOR pathway-related proteins detected by Western blot. Data were expressed as mean ± SD. Pairwise comparisons were analyzed using independent *t* test, *N* = 5. * vs sham group, *p* < 0.05.

The SCI rat model was established and identified by BBB locomotor rating scale and IPT. BBB locomotor scores and IPT scores of rats in the SCI group were decreased compared to those in the sham group (Fig. 1H, I). Rat spinal cord tissues were extracted on the 30th day after surgery and SCI was evaluated by HE staining, which exhibited destroyed central gray matter and peripheral white matter and decreased motoneurons in anterior horn in SCI rats (Fig. 1J). TUNEL staining manifested facilitated apoptosis of spinal cord tissues in SCI rats relative to that in sham-operated rats (Fig. 1K). The above results demonstrated the successful establishment of the SCI rat model.

A total of 957 highly-expressed genes and 1120 lowly-expressed genes were identified through differential expression analysis on GSE45006 (Fig. 1A). Totally 220 candidate genes were identified through the intersections of weak DEGs and SCI-related genes among the top 3000 SCI-related genes on the GeneCards database (Fig. 1B). To screen these genes, the gene co-expression network was constructed through Coexpedia (Fig. 1C), among which 73 genes had co-expression relationship scores greater than 30. Correlation of the 73 candidate genes with SCI was analyzed using Phenolyzer (Fig. 1D) and among them, 20 genes including MAPT, CAMK2B, and EEF1A2 had higher correlation scores, the expression heatmaps of which in GSE45006 were drawn (Fig. 1E). KEGG enrichment was analyzed using KOBAS (Fig. 1F). Genes are mainly involved in the pathways including cAMP, ErbB, Rap1, and PI3K-Akt, and EEF1A2 was reported to activate the Akt/mTOR pathway [21]. Our result showed that EEF1A2 was poorly expressed in SCI (Fig. 1G). Expressions of EEF1A2, p-Akt/Akt, and p-mTOR/mTOR were decreased in SCI rats (Fig. 1L). Briefly, EEF1A2 was weakly expressed and Akt/mTOR pathway was repressed in SCI rats.

### EEF1A2 overexpression inhibited SCI progression in rats

**Fig. 2:**
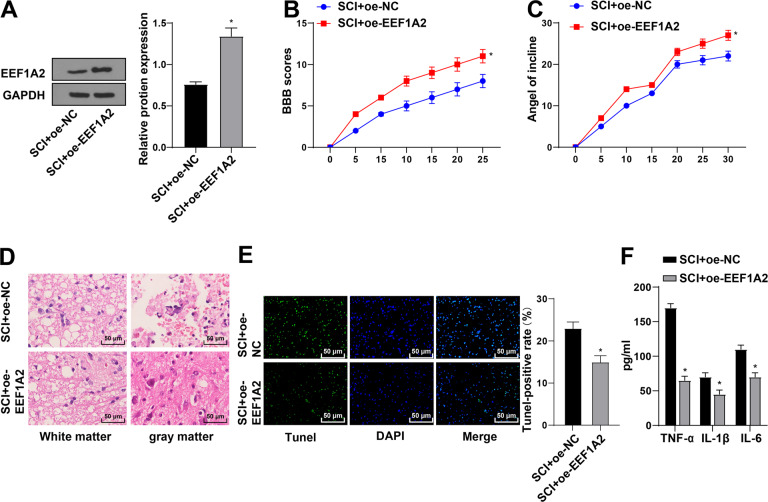
EEF1A2 overexpression inhibited SCI progression in rats. **A** expression of EEF1A2 in spinal cord tissues detected by Western blot; (**B**) locomotor ability detected by BBB locomotor rating scale; (**C**) neuronal function detected by inclined plane test; (**D**) pathological conditions of spinal cord assessed by HE staining; (**E**) apoptosis of spinal cord tissues detected by TUNEL staining; (**F**) expressions of TNF-α, IL-1β and IL-6 detected by ELISA. Data were expressed as mean ± SD and analyzed using independent *t* test, *N* = 5. * vs SCI + oe-NC group, *p* < 0.05.

EEF1A2 was then overexpressed in SCI rats to further investigate its role in SCI. EEF1A2 was increased in the SCI + oe-EEF1A2 group compared with that in the SCI + oe-NC group (Fig. 2A). Compared with the SCI + oe-NC group, the SCI + oe-EEF1A2 group showed increased BBB locomotor score and IPT scores (Fig. 2B, C), improved pathological conditions, increased amount of motoneurons in anterior horn (Fig. 2D), decreased apoptosis of spinal cord tissues (Fig. 2E), and decreased levels of TNF-α, IL-1β, and IL-6 (Fig. 2F). Altogether, EEF1A2 overexpression inhibited SCI progression in rats.

### EEF1A2 overexpression stimulated viability and limited apoptosis of H/R-treated spinal cord neurons

**Fig. 3:**
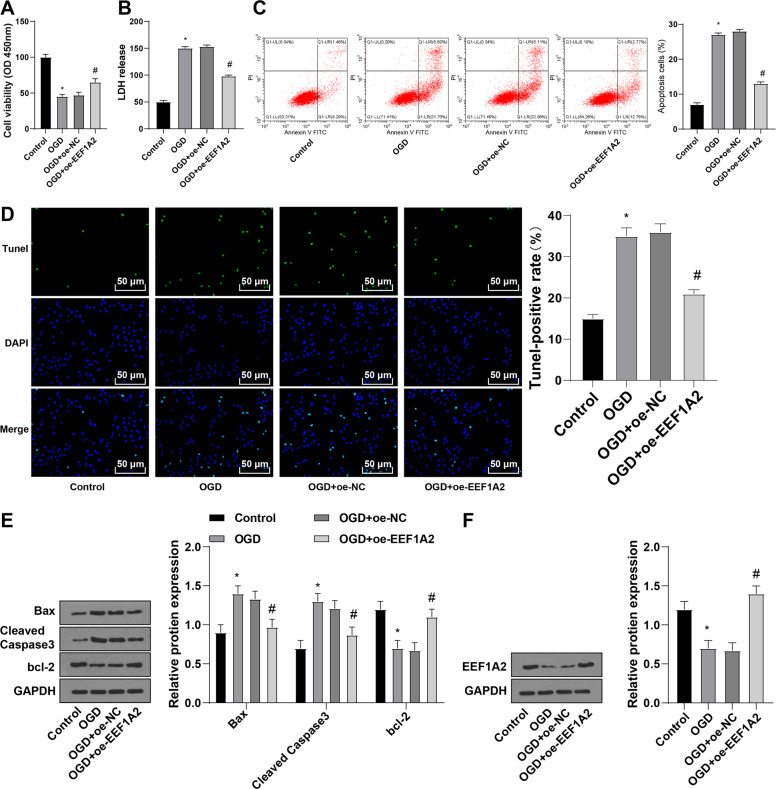
EEF1A2 overexpression promoted viability and inhibited apoptosis of H/R-treated spinal cord neurons. **A** neuronal viability detected by CCK-8; (**B**) cell toxicity detected by LDH release assay; (**C**) neuronal apoptosis detected by flow cytometry; (**D**) neuronal apoptosis detected by TUNEL staining; (**E**) expressions of Bax, cleaved caspase3, and bcl-2 detected by Western blot; (**F**) expression of EEF1A2 detected by Western blot. Data were expressed as mean ± SD and analyzed using independent *t* test. The experiment was repeated three times. * vs control group, *p* < 0.05; # vs OGD + oe-NC group, *p* < 0.05.

To investigate the effect of EEF1A2 on SCI, primary rat spinal motoneurons were treated with H/R. Compared to the control group, the OGD group exhibited decreased neuronal viability (Fig. 3A), increased LDH release (Fig. 3B), enhanced neuronal apoptosis (Fig. 3C, D), and augmented Bax expression and amounts of cleaved caspase 3 along with decreased Bcl-2 expression (Fig. 3E). Overall, H/R treatment inhibited viability and promoted apoptosis of spinal cord neurons.

To clarify the effect of EEF1A2 on H/R-treated rat spinal cord neurons, EEF1A2 expression was detected by Western blot. EEF1A2 expression was decreased in the OGD group relative to that in the control group and increased in the OGD + oe-EEF1A2 group relative to that in the OGD + oe-NC group (Fig. 3F). Enhanced cell viability and Bcl-2 expression and decreased LDH release, neuronal apoptosis, Bax expression, and amounts of cleaved caspase 3 were found in the OGD + oe-EEF1A2 group compared to those in the OGD + oe-NC group (Fig. 3A-E). Collectively, EEF1A2 overexpression promoted viability and suppressed apoptosis of spinal cord neurons.

### METTL14 downregulated EEF1A2 in SCI by mediating m6A methylation of EEF1A2

**Fig. 4:**
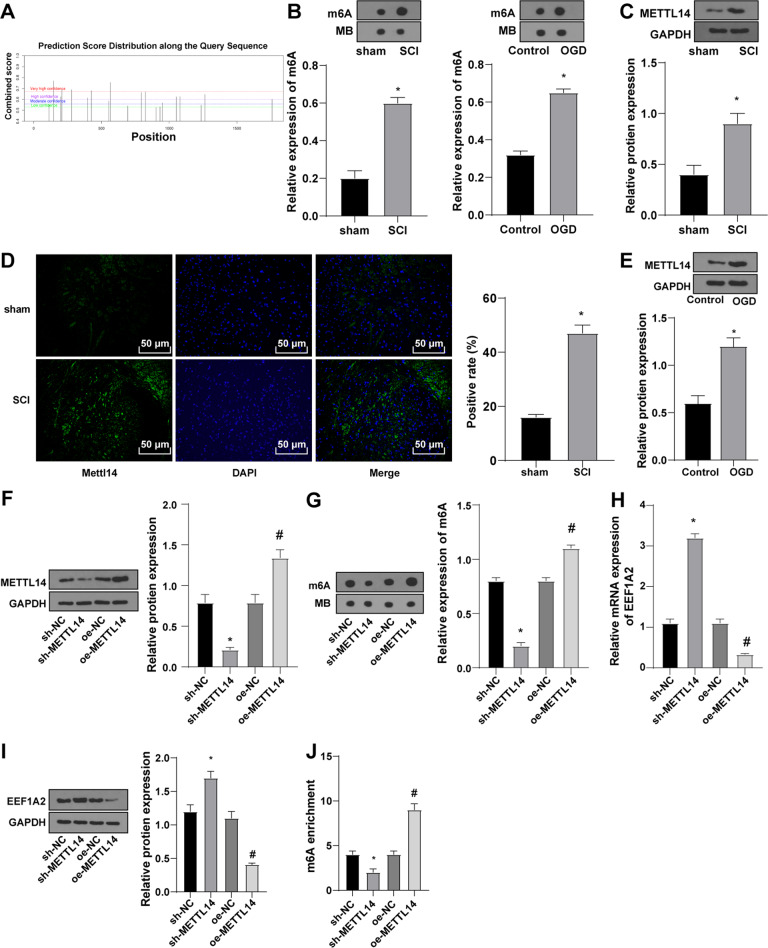
Mettl14 downregulated EEF1A2 in SCI by mediating m6A methylation of EEF1A2. **A** possible m6A methylation sites of EEF1A2; (**B**) m6A level in spinal cord tissues and H/R neurons detected by Dot blot assay; (**C**) expression of Mettl14 in rat spinal cord tissues detected by Western blot (*N* = 5); (**D**) expression of Mettl14 in spinal cord tissues detected by immunofluorescent staining (*N* = 5); (**E**) expression of Mettl14 in H/R neurons detected by Western blot; (**F**): expression of Mettl14 detected by Western blot; (**G**) total m6A level detected by Dot blot assay; (**H**) mRNA expression of EEF1A2 detected by RT-qPCR; (**I**) EEF1A2 protein expression detected by Western blot; (**J**) m6A modification level of EEF1A2 detected by Me-RIP assay. Data were expressed as mean ± SD and analyzed using independent *t* test. The experiment was repeated three times. * vs sham group, control group, sh-NC group, *p* < 0.05; # vs oe-NC group, *p* < 0.05.

m6A2Target database predicted that METTL14 might work on EEF1A2 and regulate its downstream methylation (Table 1). METTL14 and m6A were elevated in spinal cord tissues in SCI, and impaired motor function was restored and neuronal apoptosis was decreased after silencing METTL14 [22]. SRAMP database predicted that EEF1A2 might be modified by m6A methylation (Fig. 4A). m6A level was increased in SCI rats and the OGD group (Fig. 4B). METTL14 level was increased in spinal cord tissues of rats in the SCI group (Fig. 4C, D) and neurons in the OGD group (Fig. 4E). METTL14 was subsequently silenced or overexpressed. METTL14 expression and m6A level were reduced in the sh-METTL14 group relative to those in the sh-NC group and elevated in the oe-METTL14 group relative to those in the oe-NC group (Fig. 4F, G). Briefly, METTL14 may modify m6A methylation of EEF1A2. EEF1A2 level was increased in the sh-METTL14 group relative to that in the sh-NC group and decreased in the oe-METTL14 group relative to that in the oe-NC group (Fig. 4H, I). m6A modification level of EEF1A2 was decreased in the sh-METTL14 group relative to that in the sh-NC group and increased in the oe-METTL14 group relative to that in the oe-NC group (Fig. 4J). Shortly, METTL14 downregulated EEF1A2 in SCI by mediating m6A modification of EEF1A2.

**Table 1:** Binding result of searching EEF1A2 on m6A2Target database.

Class	WER name	Target gene	Interaction	Method	Downstream effect
Writer	METTL14	EEF1A2	Protein–Protein	mass spectrometry	methylation
Writer	VIRMA	EEF1A2	Protein–Protein	mass spectrometry	methylation
Reader	YTHDF1	EEF1A2	Protein–RNA	CLIP-seq	No evidence
Reader	IGF2BP3	EEF1A2	Protein–RNA	RIP-seq	No evidence
Reader	YTHDC2	EEF1A2	Protein–Protein	mass spectrometry	No evidence

*METTL14* Methyltransferase-like 14, *VIRMA* Vir-like m6A methytransferase associated, *YTHDF1* YTH N6-methyladenosine RNA binding protein 1, *IGF2BP3* Insulin-like growth factor 2 messenger RNA binding protein 3, *YTHDC2* YTH domain containing 2, *EEF1A2* Eukaryotic protein elongation factor 1 alpha 2.

### METTL14 modulated H/R-treated spinal cord neurons by mediating EEF1A2

**Fig. 5:**
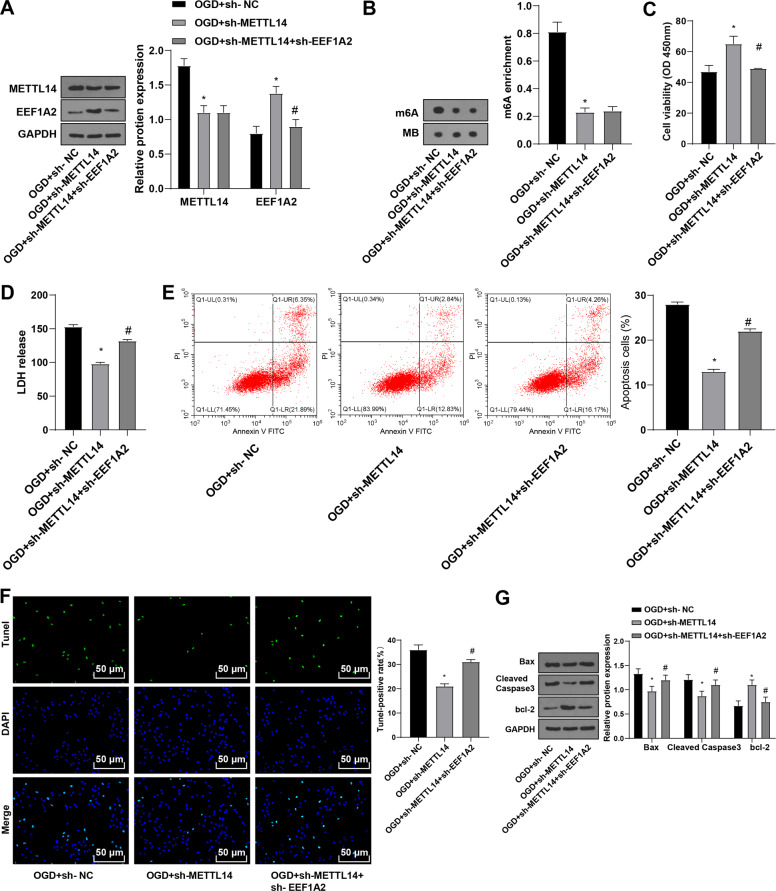
Mettl14 modulated the function of H/R-treated spinal cord neurons by mediating EEF1A2. **A** expressions of Mettl14 and EEF1A2 in neurons detected by Western blot; (**B**) total m6A level detected by Dot blot assay; (**C**) neuronal viability detected by CCK-8; (**D**) cell toxicity assessed by LDH release assay; (**E**) neuronal apoptosis detected by flow cytometry; (**F**) neuronal apoptosis detected by TUNEL staining; (**G**) expressions of Bax, cleaved caspase3 and bcl-2 detected by Western blot. Data were expressed as mean ± SD and analyzed using independent *t* test. The experiment was repeated three times. * vs OGD + sh-NC group, *p* < 0.05; # vs OGD + sh-Mettl14 group, *p* < 0.05.

To investigate the effect of METTL14-mediated EEF1A2 m6A methylation, EEF1A2 was silenced after silencing METTL14. METTL14 was decreased and EEF1A2 was increased in the OGD + sh-METTL14 group and decreased in the OGD + sh-METTL14 + sh-EEF1A2 group (Fig. 5A). m6A level was decreased in the OGD + sh-METTL14 group, without significant differences between the OGD + sh-METTL14 group and OGD + sh-METTL14 + sh-EEF1A2 group (Fig. 5B). Enhanced cell viability and Bcl-2 expression and decreased LDH release, neuronal apoptosis, Bax expression, and amounts of cleaved caspase 3 were found in the OGD + sh-METTL14 group compared to those in the OGD + sh-NC group; opposite results were shown in the OGD + sh-METTL14 + sh-EEF1A2 group relative to the OGD + sh-METTL14 group (Fig. 5C-G). Jointly, METTL14 promoted neuron apoptosis in the spinal cord via mediating m6A methylation of EEF1A2.

### METTL14 modulated SCI by mediating EEF1A2

**Fig. 6:**
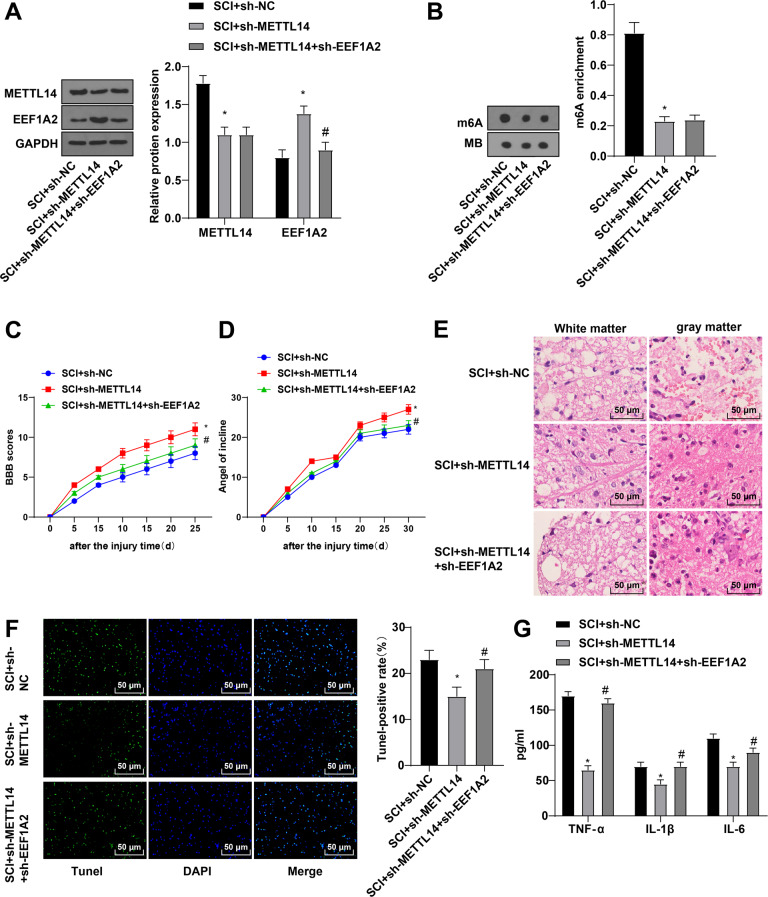
Mettl14 modulated SCI by mediating EEF1A2 expression. **A** expressions of Mettl14, EEF1A2 proteins in spinal cord tissues detected by Western blot; (**B**) total m6A level detected by Dot blot assay; (**C**) locomotor ability detected by BBB locomotor rating scale; (**D**) neuronal function detected by inclined plane test; (**E**) pathological conditions of spinal cord assessed by HE staining; (**F**) apoptosis of spinal cord tissues detected by TUNEL staining; (**G**) expressions of TNF-α, IL-1β and IL-6 detected by ELISA. Data were expressed as mean ± SD and analyzed using independent *t* test, *N* = 5. * vs SCI + sh-NC group, *p* < 0.05; # vs SCI + sh-Mettl14 + sh-EEF1A2 group, *p* < 0.05.

To study the effect of METTL14-mediated EEF1A2 m6A methylation on SCI, EEF1A2 was silenced after silencing METTL14 in SCI rats. METTL14 was decreased, while EEF1A2 was increased in the SCI + sh-METTL14 group and decreased in the SCH + sh-METTL14 + sh-EEF1A2 group (Fig. 6A). Relative to the SCI + sh-NC group, the SCI + sh-METTL14 group showed decreased m6A level, while no significant difference between the SCI + sh-METTL14 + sh-EEF1A2 group and SCI + sh-METTL14 group was observed (Fig. 6B). Compared with the SCI + sh-NC group, the SCI + sh-METTL14 group displayed increased BBB locomotor scores and IPT scores, improved pathological conditions, increased amounts of motoneurons in the anterior horn, decreased apoptosis and expressions of TNF-α, IL-1β, and IL-6, whereas further sh-EEF1A2 treatment averted the above-mentioned results (Fig. 6C–G). Collectively, METTL14 inhibited neuronal apoptosis in the spinal cord through the mediation of EEF1A2 m6A methylation in SCI.

### EEF1A2 promoted viability and limited apoptosis of spinal cord neurons by activating Akt/mTOR pathway

**Fig. 7:**
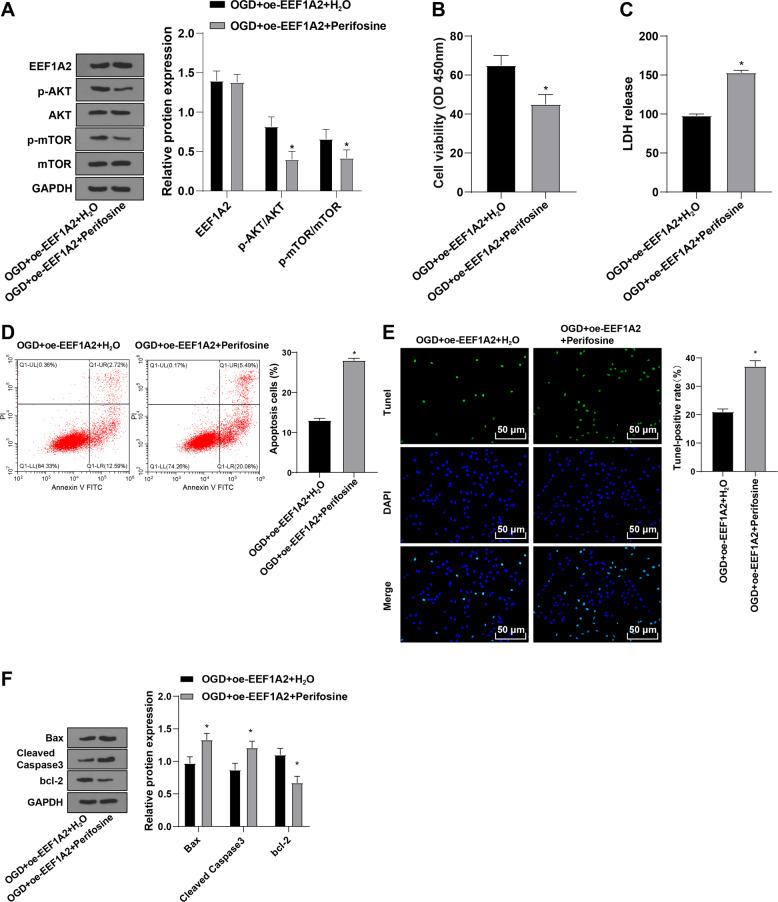
EEF1A2 promoted viability and inhibited apoptosis of spinal cord neurons by activating Akt/mTOR pathway. **A** expressions of EEF1A2 and Akt/mTOR pathway-related proteins detected by Western blot; (**B**) neuronal viability detected by CCK-8; (**C**) cell toxicity assessed by LDH release assay; (**D**) neuronal apoptosis detected by flow cytometry; (**E**) neuronal apoptosis detected by TUNEL staining; (**F**) expressions of Bax, cleaved caspase3 and bcl-2 detected by Western blot. Data were expressed as mean ± SD and analyzed using independent *t* test. The experiment was repeated three times. * vs OGD + oe-EEF1A2 + H2O group, *p* < 0.05.

To study whether EEF1A2 affects spinal cord neuron function via the Akt/mTOR pathway, neurons were treated with 40 μm Akt inhibitor (Perifosine) after overexpressing EEF1A2 [23]. Relative to the OGD + oe-EEF1A2 + H_2_O group, the OGD + oe-EEF1A2 + Perifosine group showed no significant difference of EEF1A2 and decreased expressions of p-Akt and p-mTOR (Fig. 7A), decreased neuronal viability (Fig. 7B), increased LDH release (Fig. 7C); enhanced neuronal apoptosis (Fig. 7D, E), augmented expressions of Bax and amounts of cleaved caspase 3, and diminished Bcl-2 expression (Fig. 7F). Briefly, EEF1A2 overexpression promoted viability and restrained apoptosis of spinal cord neurons by activating the Akt/mTOR pathway.

### EEF1A2 affected SCI by activating the Akt/mTOR pathway

**Fig. 8:**
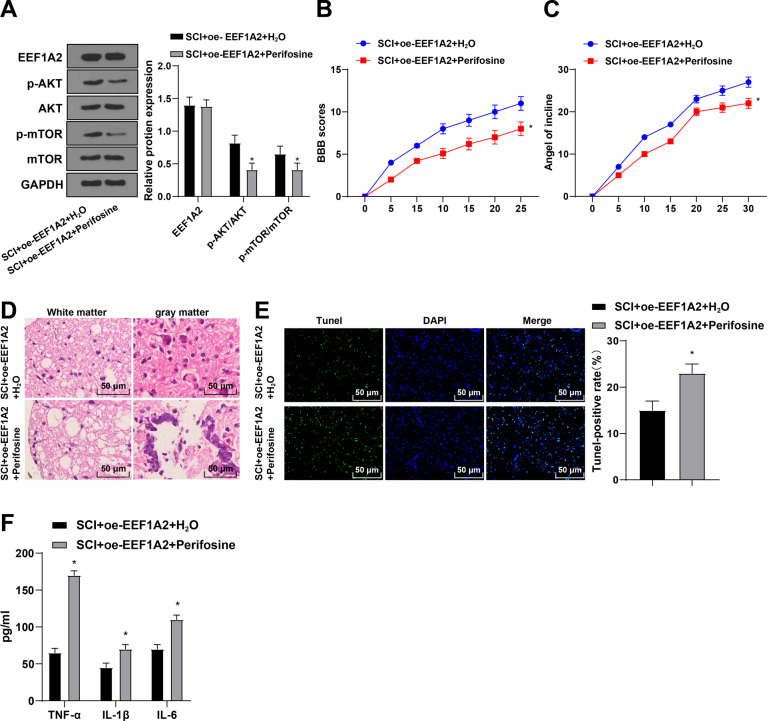
EEF1A2 affected SCI by activating the Akt/mTOR pathway. **A** expressions of EEF1A2 and Akt/mTOR pathway-related proteins in spinal cord tissues detected by Western blot; (**B**) locomotor ability detected by BBB locomotor rating scale; (**C**) neuronal function detected by inclined plane test; (**D**) pathological conditions of spinal cord assessed by HE staining; (**E**) apoptosis of spinal cord tissues detected by TUNEL staining; (**F**) expressions of TNF-α, IL-1β and IL-6 detected by ELISA. Data were expressed as mean ± SD and analyzed using independent *t* test, *N* = 5. * vs SCI + oe-EEF1A2 + H2O group, *p* < 0.05.

SCI rats were treated with 50 mg/kg (i.p.) Akt inhibitor (Perifosine) after overexpressing EEF1A2 [24]. Relative to SCI + oe-EEF1A2 + H_2_O group, the SCI + oe-EEF1A2 + Perifosine group showed no significant difference of EEF1A2 and decreased expressions of p-Akt and p-mTOR (Fig. 8A), decreased BBB locomotor score and IPT scores (Fig. 8B, C), exacerbated pathological conditions and decreased amounts of motoneurons in anterior horn (Fig. 8D), enhanced apoptosis (Fig. 8E), and increased levels of TNF-α, IL-1β, and IL-6 (Fig. 8F). Altogether, EEF1A2 overexpression inhibited SCI progression by activating the Akt/mTOR pathway in rats.

## Discussion

SCI is associated with a reduction of life expectancy and significant societal costs [25, 26]. SCI is characterized by primary injuries and secondary injuries, whereas secondary injuries including neuronal apoptosis are reversible and can be treated [27]. METTL14 regulates cell cycle progression of human cortical neural progenitor cells [28]. The current study elicited that silencing METTL14 reduced spinal cord neuron apoptosis and mitigated SCI by inhibiting m6A methylation of EEF1A2 and activating the Akt/mTOR pathway.

The SCI rat model was initially established. Next, 20 genes with high correlation scores with SCI were identified, among which EEF1A2 can activate the Akt/mTOR pathway [21]. Our results showed decreased expressions of EEF1A2, p-Akt/Akt, and p-mTOR/mTOR in SCI rats. In line with our findings, a prior study documented that EEF1A2 knockdown was found in mice featured by vacuolar degeneration of anterior horn neurons of spinal cord [29]. The activity of the PI3K/Akt/mTOR pathway is decreased in SCI rats [30]. Our results showed weakly expressed EEF1A2 and repressed Akt/mTOR pathway in SCI rats. Subsequently, EEF1A2 was overexpressed to study the effect of EEF1A2 on SCI. Our result showed that EEF1A2 expression was increased in SCI rats after EEF1A2 overexpression. Motor function is radically impaired in SCI [31]. Also, our results exhibited increased BBB locomotor scores and IPT scores in SCI rats after EEEF1A2 overexpression. The pathological conditions of spinal cord tissues were improved and anterior horn motoneurons were increased, and apoptosis in SCI rats was inhibited after EEF1A2 overexpression. Moreover, recent evidence has insinuated the levels of inflammatory cytokines are elevated in spinal cord tissues after SCI [32]. In accordance, levels of TNF-α, IL-1β, and IL-6 were decreased in SCI rats after EEF1A2 overexpression. EEF1A2 overexpression promotes cell proliferation and inhibited apoptosis in acute myeloid leukemia [33]. Consistently, our results demonstrated that EEF1A2 overexpression inhibited SCI progression in rats. To explore the effect of EEF1A2 on SCI in vitro, primary spinal cord neurons were cultured and treated with H/R. H/R treatment inhibited neuronal viability and promoted apoptosis. Furthermore, EEF1A2 was overexpressed to clarify the effect of EEF1A2 on H/R-treated spinal motoneurons. Our results showed that EEF1A2 was downregulated in OGD-treated neurons but upregulated upon EEF1A2 overexpression. It is noteworthy that silencing EEF1A2 decreases viability and increases apoptosis in Parkinson’s disease [34]. Alternatively, our results showed that neuronal viability was increased while LDH release and neuronal apoptosis were decreased in OGD-treated neurons after EEF1A2 overexpression. EEF1A2 is associated with suppressed apoptosis [35]. Specifically, EEF1A2 overexpression promoted viability and inhibited apoptosis of spinal cord neurons.

METTL14 was predicted to act on EEF1A2 and regulate its downstream methylation. Recent evidence has supported that METTL14 is essential in regulating striatal function and learning [14]. The SRAMP database predicted that EEF1A2 might be modified by m6A methylation. Previous studies have noted that m6A RNA methylation profiling was altered in SCI [36]. METTL14 is upregulated in human and rat calcified arteries [37]. Our result showed increased m6A and METTL14 levels in spinal tissues of SCI rats and OGD-treated neurons. METTL14 deletion reduces the m6A methylation modification in atherosclerosis [38]. Similarly, the overall m6A level was decreased after silencing METTL14 and increased after overexpressing METTL14. Hence, we hypothesized that METTL14 modified m6A methylation of EEF1A2. EEF1A2 was increased after silencing METTL14 and decreased after METTL14 overexpression; m6A modification level of EEF1A2 was decreased after silencing METTL14 and increased after METTL14 overexpression. METTL14 suppressed YAP1 by increasing the methylated level of YAP1 in renal ischemic reperfusion injury [39]. Altogether, METTL14 downregulated the EEF1A2 expression by mediating the m6A modification of EEF1A2.

Then, EEF1A2 and METTL14 were silenced to explore the effect of METTL14-mediated EEF1A2 m6A methylation. EEF1A2 was downregulated in OGD-treated neurons and SCI rats after silencing METTL14 and EEF1A2. The total m6A level showed no significant difference after silencing METTL14 and EEF1A2. METTL14 overexpression reduces cell viability in human nucleus pulposus cells [40]. In vitro experiments demonstrated that neuronal viability was inhibited, and LDH release and apoptosis were promoted after silencing METTL14 and EEF1A2. In vivo experiments showed decreased BBB locomotor scores and IPT scores of SCI rats, exacerbated pathological conditions, decreased motoneurons in the anterior horn, and increased apoptosis and inflammation after silencing METTL14 and EEF1A2. METTL14 overexpression promotes apoptosis and enhances m6A level in cisplatin-treated HK-2 cells [41]. EEF1A2 deficiency induces apoptosis in MPP^+^-treated SH-SY5Y cells by activating caspase3 [34]. Overall, METTL14 promoted spinal cord neuron apoptosis in SCI via mediating EEF1A2 m6A methylation.

The PI3K/Akt/mTOR pathway participates in spinal cord neuron apoptosis in SCI [42]. EFF1A2 is associated with the PI3K/Akt/mTOR pathway [20]. To investigate whether EEF1A2 affects the spinal cord neurons via the Akt/mTOR pathway, neurons were treated with an Akt inhibitor after overexpressing EEF1A2. Our results showed that expressions of p-Akt and p-mTOR were decreased while EEF1A2 showed no significant difference in OGD-treated neurons after EEF1A2 overexpression and Perifosine treatment. The Akt activator SC79 protects cell viability in rotenone-injured SH-SY5Y cells [43]. Neuronal viability was decreased while LDH release and apoptosis were increased in OGD-treated neurons after EEF1A2 overexpression and Perifosine treatment. The Akt/mTOR pathway is essential in the protection against cadmium-induced neuronal apoptosis [44]. The PI3K/Akt/mTOR pathway elevates Bcl-2 expression but reduces expressions of Bax and amounts of cleaved caspase 3 in ischemia-reperfusion injury [45]. Expressions of Bax and cleaved caspase 3 were increased while Bcl-2 expression was decreased in OGD-treated neurons after EEF1A2 overexpression and Perifosine treatment. EFF1A is pivotal in 6-OHDA-induced neurodegeneration via the PI3K/Akt/mTOR pathway [46]. Fangji decoction represses hippocampal neuron apoptosis by regulating the Akt/mTOR pathway in febrile seizures [47]. These data further confirmed that EEF1A2 overexpression promoted viability and suppressed spinal cord neuron apoptosis by activating the Akt/mTOR pathway. Furthermore, SCI rats were treated with Perifosine after EEF1A2 overexpression to explore the effect of EEF1A2 through the Akt/mTOR pathway on SCI. Melatonin promotes locomotor recovery of motoneuron in the anterior horn in SCI through the PI3K/Akt/mTOR pathway [48]. In vivo experiment showed decreased BBB locomotor scores and IPT scores and motoneurons in anterior horn and increased apoptosis and levels of TNF-α, IL-1β, and IL-6 in SCI rats with EEF1A2 overexpression and Perifosine. The decrease in PI3K/Akt/mTOR pathway is associated with increases in pro-inflammatory cytokines in the rat cortex and hippocampus [49]. EEF1A2 is correlated with the pathological process of SCI [50]. The Akt/mTOR/p70S6K pathway activation facilitates the recovery of motor function in SCI [51]. Collectively, EEF1A2 overexpression impeded SCI progression by activating the Akt/mTOR pathway.

In conclusion, silencing METTL14 suppressed spinal cord neuron apoptosis in SCI via mediating EEF1A2 m6A methylation and the Akt/mTOR pathway, and thus providing novel insights into the management of SCI. Despite the findings, this study has some limitations. The downstream targets of METTL14 and other possible pathways were not investigated in this study. We will perform in-depth analysis in the future to enrich and support this study.

## Material and methods

### Bioinformatics analysis

SCI-related chip GSE45006 was searched on the GEO database (https://www.ncbi.nlm.nih.gov/geo/), including 4 sham samples and 20 SCI samples. The annotation file was downloaded from the platform GPL1355 [Rat230_2] Affymetrix Rat Genome 230 2.0 Array. Differential gene expression analysis was conducted using the R-language limma package (http://www.bioconductor.org/packages/release/bioc/html/limma.html) with |log2FC| >1 and *p* value <0.05 as criteria to screen the differentially expressed genes (DEGs). Differential gene expression heatmap was graphed using the R-language pheatmap package (https://cran.r-project.org/web/packages/pheatmap/index.html). SCI-related genes were searched on the GeneCards database (https://www.genecards.org/). The overlapping weak DEGs with SCI-related genes were searched using jvenn (http://jvenn.toulouse.inra.fr/app/example.html). The co-expression relationships among genes were analyzed using Coexpedia (http://www.coexpedia.org/). Genes with co-expression relationship score greater than 30 were further analyzed. The correlation between genes and SCI was analyzed using Phenolyzer (http://phenolyzer.wglab.org/). The top 20 genes ranked by correlation scores were investigated further. KEGG enrichment analysis of genes was conducted using KOBAS (http://kobas.cbi.pku.edu.cn/kobas3). The methyltransferase of the key factors was predicted on the m6A2Target database (http://m6a2target.canceromics.org/#/search). m6A methylation was predicted on the SRAMP database (http://www.cuilab.cn/sramp).

### Animal model

SCI rat model was established using a total of 40 specific-pathogen-free Sprague-Dawley rats (male, aged 8 weeks, weighing 200 ± 20 g; Hunan SJA Laboratory Animal Co., Ltd., Changsha, China).

SCI rat model was treated in conformity with reference [52]. Specifically, rats were anesthetized with an intraperitoneal injection of 4% pentobarbital sodium (35 mg/kg) after weight measurement. To expose the posterior vertebral arch from T8 to T12, an incision was subsequently made on the skin along the dorsomedial line to the aponeurosis and muscle plane. Laminectomy (3 mm) was performed from the caudal end of the T10 vertebra to the caudal end of the T11 vertebra under a dissection stereomicroscope. Infinite Horizons impactor (Infinite Horizons, L.L.C., Lexington, KY, USA) was utilized to induce contusion SCI at the force of 60 kdyn/cm [2, 22]. The incision was sutured, followed by intramuscular injection of 20000 units of penicillin once a day for three days. Incisions of rats in the sham group (*N* = 10) were sutured after skin incision without modeling surgery and related treatment. SCI rat model was established and SCI rats were assigned to the following groups (*N* = 10 per group): SCI group (SCI treatment), SCI + sh-NC group (injected with silencing negative control lentivirus after SCI treatment), SCI + sh-METTL14 + sh-EEF1A2 group (injected with silencing EEF1A2 and silencing METTL14 lentivirus after SCI treatment), SCI + oe-NC group (injected with overexpressed EEF1A2 NC lentivirus after SCI treatment), SCI + oe-EEF1A2 group (injected with overexpressed EEF1A2 lentivirus after SCI treatment), SCI + oe-EEF1A2 + H_2_O group [injected with overexpressed EEF1A2 lentivirus and treated with 50 mg/kg (i.p.) H_2_O after SCI treatment] and SCI + oe-EEF1A2 + Perifosine group [injected with overexpressed EEF1A2 lentivirus and treated with 50 mg/kg (i.p.) Perifosine after SCI treatment [24]]. Lentivirus treatment was conducted three days following laminectomy (on day 0, 1, and 2). sh-NC, sh-METTL14, sh-EEF1A2, oe-NC, and oe-EEF1A2 lentivirus (50 μL/day, 100 nmoL/mL; RiboBio, Guangzhou, China) were intrathecally injected through lumbar puncture for 15 min per day [53].

### Basso, Beattie, and Bresnahan (BBB) locomotor rating scale

The locomotor function of SCI rats was evaluated using BBB locomotor rating scale with scores ranging from 0 to 21. A score of 0 represented no visible movement of the hindlimb (complete paralysis) while a score of 21 represented complete mobility. Rats were placed in the open field for 5 min each day on the 0th, 5th, 10th, 15th, 20th, 25th, and 30th days post-surgery. The four-min evaluation on each rat was made and scores of hindlimb movement were recorded by three experienced researchers. The average score was the final score [54].

### Inclined plane test (IPT)

In IPT, rats were placed on the smooth inclined plane. The angle of the inclined plane started from a horizontal position (0°) and increased by 5–10° each time. The maximum angle at which rats remained on the plane for 10 s was recorded [55].

### Hematoxylin and eosin (HE) staining

Rats were euthanized by intraperitoneally injecting excessive pentobarbital sodium on the 30th day post-surgery. Spinal cord tissues (2 cm in length) centering at the injured spinal cord were extracted and embedded after opening the spine. The paraffin-embedded tissues were sliced, dewaxed, hydrated, stained with hematoxylin (PT001; Bogoo Biotechnology, Shanghai, China) for 10 min, and rinsed with running water for 30–60 s, followed by differentiation with 1% hydrochloric ethanol for 30 s and rinsing with running water for 5 min. The slices were then stained with eosin (0001-H; Beijing Xinhua Lvyuan Technology Co., Ltd., Beijing, China) for 1 min, dehydrated with gradient ethanol (at the concentration of 70%, 80%, 90%, 95%, and 100%) for 1 min in each concentration, cleared with carbonate xylene for 1 min and xylene I and II (GD-RY1215-12; Shanghai Guduo Biological Technology Co., Ltd., Shanghai, China) twice (1 min each time), and sealed with neutral gum in the airing chamber. Morphological changes in spinal cord tissues were photographed and observed under a Zeiss fluorescence microscope (PrimoStariLED; Bioresearch, Beijing, China).

### TUNEL staining

TUNEL staining was adopted to evaluate the apoptosis of spinal cord tissues. Briefly, paraffin-embedded slices in different groups were dewaxed, hydrated and cells were permeated and added with TUNEL staining reaction mixture (Roche, Shanghai, China). A fluorescence microscope (#Eclipse Ti-U; Nikon, Tokyo, Japan) was utilized to count and photograph the apoptotic cells.

### Western blot

Tissues were collected with the trypsin digestion method and lysed with enhanced radio immunoprecipitation assay lysis buffer (Boster, Wuhan, China) containing protease inhibitor. Protein concentration was determined using the BCA protein quantitative kits (Boster). Subsequently, the protein samples were separated with 10% sodium dodecyl sulfate-polyacrylamide gel electrophoresis (SDS-PAGE) and transferred onto polyvinylidene fluoride (PVDF) membranes. The membranes were blocked with 5% bovine serum albumin (BSA) at room temperature for 2 h to reduce non-specific binding. The membranes were added with diluted primary antibodies EEF1A2 (ab227824, 1:1000; Abcam, Cambridge, MA, USA), METTL14 (ab252562, 1:1000; Abcam), p-Akt (ab8933, 1:500; Abcam), Akt (ab8805, 1:500; Abcam), p-mTOR (ab109268, 1:1000; Abcam), mTOR (ab134903, 1:500; Abcam), cleaved caspase 3 (ab32042, 1:1000; Abcam), Bax (ab32503, 1:1000; Abcam), Bcl-2 (ab32124, 1:1000; Abcam), and GAPDH (ab9485, 1:2500; Abcam) and incubated at 4 °C overnight. After washing the membranes, HRP-conjugated goat anti-rabbit secondary antibody (ab6721, 1:2000; Abcam) was added and incubated for 1 h. ECL working solution (EMD Millipore, Billerica, MA, USA) was added and incubated with membranes for 1 min. After removing excess ECL working solution, the membranes were sealed with plastic films and placed in a dark box for X-ray film exposure for 5–10 min before developing and fixing. The gray value of each band in the images was quantified with Image J analysis software (NIH, Bethesda, MD, USA) with GAPDH as an internal control.

### Reverse transcription-quantitative polymerase chain reaction (RT-qPCR)

The total RNA was extracted using TRIzol reagent (15596026; Invitrogen, Carlsbad, CA, USA). RNA was reverse-transcribed into cDNA following the instructions of PrimeScript RT reagent kit (RR047A; Takara, Tokyo, Japan). RT-qPCR was performed on the synthesized cDNA with Fast SYBR Green PCR kit (ABI, Foster City, CA, USA) and ABI PRISM 7300 RT-PCR system (ABI) with three repetitions per well. Relative expressions of each gene were analyzed adopting the 2^−ΔΔCt^ method with GADPH as an internal control. Primer sequences are illustrated in Table 2.

**Table 2:** Primer sequences.

Name of primers	Sequences
EEF1A2 (PMID: 33859743)	F 5- GGCCACCTCATCTACAAATG -3
	R 5- TCGAACTTCCAGAGGGAGAT -3
GAPDH	F 5- GTGGACCTGACCTGCCGTCT -3
	R 5- GGAGGAGTGGGTGTCGCTGT -3

*EEF1A2* Eukaryotic protein elongation factor 1 alpha 2, *GAPDH* Glyceraldehyde-3-phosphate dehydrogenase, *F* Forward, *R* Reverse.

### Immunofluorescence staining

The climbing slides were fixed with 4% paraformaldehyde for 15 min, rinsed with phosphate buffer saline (PBS) 3 times, and dried with absorbent paper. The slides were dripped with normal goat serum and blocked for 30 min. The blocking solution was dried with absorbent paper and each slide was dripped with primary antibody METTL14 (ab252562, 1:200; Abcam) and incubated at 4 °C overnight without washing beforehand. The slides were rinsed thrice with PBS (3 min each time) and dripped with Alexa Fluor 488-labeled goat anti-rabbit IgG (ab150077; Abcam) and Alexa Fluor 647-labeled goat anti-rabbit IgG (150083; Abcam) and incubated for 1 h at 37 °C avoiding light exposure. After 3 times of PBS washes in the dark, the slides were stained with 5 μg/mL 4’,6-diamidino-2-phenylindole (DAPI) for 5 min, washed 3 times with PBS (5 min each time), sealed at 4 °C avoiding light, and observed under laser confocal microscope (Carl Zeiss, Jena, Germany) using NIS-Elements Viewer software.

### Culture and transfection of primary spinal cord neurons

On E13 day (E0 = the 1st day after mating) of mating, the pregnant animal was euthanized using CO_2_. Embryos were isolated from the uterus and immediately placed in the cold buffer. The spinal cord of the embryo was dissected and incubated at 37 °C, detached with 0.15% trypsin for 25 min, rinsed at room temperature, and centrifuged for 4 min at 200 × g. The spinal cord tissues were resuspended in a solution containing Dnase and trypsin inhibitor. The collected supernatant was centrifuged for 4 min at 200 × g for 15 min to obtain cells. Cell purity was assessed by immunofluorescence staining with neuronal marker NeuN. Specifically, 90% positive staining for NeuN in primary spinal cord neurons indicated that the purity of neurons was above 90% and the neurons were available for following experimentation. Cells were cultured with neuralbasal growth medium (2% NS21, 1% HEPES, and 1% l-glutamine) with 95% humidity and 5% CO_2_ at 37 °C. Lentivirus plasmid and virus packaging kit were purchased from GeneCopoeia (Rockville, MD, USA). After 48 h of co-transfection into HEK293T cells with lentivirus transfection kit, viral titer was assessed using p24 ELISA kit (Cell Biolabs, San Diego, CA, USA). Then, neurons were infected with prepared lentivirus particles and cultured for 24 h, and treated with oxygen-glucose deprivation (OGD) based on reference [56]. Briefly, after three gentle rinses with PBS, neurons were placed in the medium added with sugar-free Dulbecco’s modified Eagle’s medium (DMEM) pre-heated to 37 °C and cultured in an anaerobic incubator for 2 h with 95% N_2_ and 5% CO_2_ at 37 °C and then the normal culture was resumed. Neurons were divided into nine groups: control group (normal culture), OGD group [treated with hypoxia/reoxygenation (H/R)], OGD + oe-NC group (treated with H/R and transfected with overexpressed NC), OGD + oe-METTL14 group (treated with H/R and transfected with METTL14 overexpression), OGD + sh-NC group (treated with H/R and transfected with corresponding silencing NC), OGD + sh-METTL14 group (treated with H/R and transfected with the lentivirus of silencing METTL14), OGD + sh-METTL14 + sh-EEF1A2 group (treated with H/R and transfected with lentivirus of silencing METTL14 and silencing EEF1A2), OGD + oe-EEF1A2 + H_2_O group (treated with H/R and H_2_O and transfected with lentivirus of overexpressed EEF1A2), and OGD + oe-EEF1A2 + Perifosine group [treated with H/R, transfected with lentivirus of overexpressed EEF1A2 and treated with 40 μM Akt inhibitor (Perifosine) [23]].

### Cell Counting Kit-8 (CCK-8)

Cell viability was detected using CCK-8 kits (CK04; Dojindo Molecular Technologies, Kumamoto, Japan). Neurons at the logarithmic growth phase were seeded in 96-well plates (1 × 10^4^ cells/well) and pre-cultured for 24 h and transfected accordingly. Following the 48-h transfection, neurons were added with 10 μL CCK-8 reagent and incubated at 37 °C for 3 h. Absorbance at 450 nm was detected with a microplate reader.

### Lactate dehydrogenase (LDH) release assay

Following 24 h of transfection, LDH release was detected using LDH Cytotoxicity Assay kits (Beyotime, Shanghai, China) in strict conformity with the instructions of the kit. Absorbance peak at an optical density of 490 nm was detected.

### Flow cytometry

Following 48 h of transfection, cells were detached by 0.25% trypsin (EDTA-free) (YB15050057; Shanghai Yubo Biotechnology Co., Ltd., Shanghai, China), collected into flow tubes and centrifuged to discard the supernatant. After three washes with cold PBS, cells were centrifuged and the supernatant was removed. Cell apoptosis was detected in line with the instructions of Annexin-V-FITC Apoptosis Detection kit (K201-100; BioVision, San Francisco, CA, USA). Subsequently, 100 μL staining solution (1 × 10^6^ cells) was used to resuspend cells and mixed thoroughly. The 525 nm and 620 nm band-pass filters were excited at 488 nm to detect FITC and PI fluorescence, followed by apoptosis detection [57].

### TUNEL assay

Cells at the logarithmic phase were collected and seeded in the cover slides in six-well plates at 1 × 10^6^ cells/mL after 48 h of transfection. TUNEL apoptosis was detected using In Situ Cell Death Detection kit [11684795910; Roche, Basel, Switzerland (green fluorescence)]. Cell suspension solution was applied to the cover slides and fixed for 1 h with 4% paraformaldehyde. The cells were then treated with 0.1% Triton X-100 (Beyotime) for 3 min at 4 °C, incubated with 50 μL TUNEL solution at 37 °C for 1 h without light exposure, and washed with PBS three times. Fluorescence intensity was observed under a fluorescence microscope after sealing cells with an anti-fluorescence quenching solution. Finally, 5 high power fields (400×) were randomly selected to count the number of TUNEL-positive cells [58].

### Methylated RNA immunoprecipitation (Me-RIP)

The total RNA was extracted using the TRIzol method. The purified mRNA was isolated using PolyATtract® mRNA Isolation Systems (A-Z5300; A & D Technology Corporation, Beijing, China). Anti-m6A antibody (ab151230, 1:500, Abcam) or anti-IgG antibody (ab109489, 1:100; Abcam) was added into IP buffer (20 mM Tris pH 7.5, 140 mM NaC1, 1% NP-40, 2 mM EDTA) and co-incubated with protein A/G magnetic beads for 1 h for binding. IP buffer containing Rnase inhibitor and protease inhibitor was added with purified mRNA and magnetic beads-antibody complex and incubated overnight at 4 °C. The RNA was eluted with elution buffer and purified by phenol-chloroform extraction. EEF1A2 was analyzed by RT-qPCR.

### Dot blot assay

The total RNA was extracted using the TRIzol method. The purified mRNA was isolated using PolyATtract® mRNA Isolation Systems (A-Z5300; A & D Technology Corporation). The isolated mRNA was denatured under ultraviolet (UV) irradiation for 7 min and dotted on Amersham Hybond-N membranes. The membranes were optimized for nucleic acid transfer (GE Healthcare, Boston, MA, USA). After regimen UV cross-linking, the RNA was rinsed with 1 × phosphate-buffered saline Tween-20 (PBST) for 5 min, blocked with 5% skim milk powder, and incubated with anti-m6A antibody overnight at 4 °C. Finally, the membranes were visualized using the Chemilum HRP Substrate (Immobilon Western, Millipore, Bedford, MA, USA).

### Statistical analysis

All the data were processed using SPSS 21.0 statistical software (IBM Corp., Armonk, NY, USA). Measurement data were presented as mean ± standard deviation. Normality and homogeneity of variance were verified, which were in consistency with normal distribution and homogeneity of variance. Pairwise comparisons were analyzed using unpaired *t* test while comparisons among multiple groups were analyzed using one-way analysis of variance (ANOVA) or repeated measure ANOVA, followed by Turkey’s post-test. A value of *p* < 0.05 was regarded statistically significant.

## Data Availability

All the data generated or analyzed during this study are included in this published article.
